# Psychological Interventions for Healthcare Providers With PTSD in Life-Threatening Pandemic: Systematic Review and Meta-Analysis

**DOI:** 10.3389/fpsyt.2021.697783

**Published:** 2021-07-29

**Authors:** Zeyuan Sun, Chuan Yu, Yue Zhou, Zhenmi Liu

**Affiliations:** ^1^West China School of Public Health, Sichuan University, Chengdu, China; ^2^Harvard Law School, Harvard University, Cambridge, MA, United States

**Keywords:** PTSD, healthcare provider, systematic review, meta-analysis, COVID-19

## Abstract

**Objective:** This study aims to evaluate the effect of psychological interventions on healthcare providers (HCP) with post-traumatic stress disorder (PTSD) due to their necessary exposure in life-threatening pandemic.

**Methods:** We performed a systematic research on Medline, Embase, Cochrane Central, PsycInfo, Cochrane Central Register of Controlled Trials, Clinicaltrials.gov, ProQuest PTSD Pubs ProQuest Dissertations & Theses Global, and other gray databases by January 2021. Randomized controlled trials involving therapeutic interventions for HCP with PTSD were included. The primary outcome was PTSD symptom severity. Summary standardized mean differences (SMDs) and 95% confidence intervals were estimated using inverse variance meta-analysis with fixed effects. Risks of bias were assessed using Cochrane methods.

**Results:** Among 773 citations, this review includes six studies, randomizing 810 participants. A meta-analysis of the effect of interventions compared to placebo showed a significant reduction of PTSD symptom severity: Cognitive Behavioral Therapy-Brief (CBT-B) (*M* = 27.80, 95% CI: 17.12, 38.48), Cognitive Behavioral Therapy-Long (CBT-L) (*M* = 26.50, 95% CI: 15.75, 37.25), and Mindfulness-Based Stretching and Deep Breathing Exercise (MBX) (*M* = 17.2, 95% CI: 6.57, 27.83). CBT-L and CBT-B also showed a significant effect on depression severity.

**Conclusions:** The most effective and feasible treatment option for HCP with PTSD is still unclear, but CBT and MBX have displayed the most significant effects based on current limited evidence. Future research in this area—preferably large robust randomized controlled trials—is much needed.

## Introduction

Post-traumatic stress disorder (PTSD) refers to pathological responses to severe trauma (1). A range of mechanisms has been proposed to account for PTSD (2, 3), and the main indicator of PTSD is associated traumatic events, such as serious accidents including physical and sexual assault and abuse (4).

PTSD is a worldwide epidemic with 7–12% prevalence among the general population (5). Epidemiological research suggests that 60% of men and 50% of women experience at least one PTSD-qualifying traumatic event during their lives (6). PTSD could lead to serious consequences for the individuals involved, causing clinically significant distress, reduced day-to-day functioning, disabling symptoms and behaviors, and even suicide (7). Further damages include substance abuse and loss of quality of life (8). The social and economic cost of PTSD is also significant. In the United States alone, the cost is about $3 billion annually (7), while in United Kingdom, it is £11.687 million (9).

Typical occupational population such as healthcare providers (HCP) and medical emergency teams are reported to have a relatively high prevalence rate of PTSD (10). For example, 11% of ambulance personnel, 17% of midwives, and 22% of emergency physicians were reported to be once affected by PTSD (11, 12). When facing high-risk or fatal situations, HCP may be especially more likely to develop PTSD as demonstrated during previous epidemics: infected doctors suffered multiple mental problems and, as a result, the confidence of local residents in healthcare services had also been adversely affected (13, 14). The recent outbreak of COVID-19 has likewise put many HCP under the risk of infection and of PTSD. A recent study found that nearly 300,000 healthcare workers had been infected with COVID-19 (15). If not treated properly after developing PTSD, negative results such as burnout (16), secondary stress disorder (17), and other mental disorders would likely ensue, negatively affecting HCP and their ability to provide healthcare service (8). Unfortunately, however, interventions specifically targeting HCP to prevent and treat their PTSD remain rare (18).

Despite a growing number of available research on PTSD interventions, a few focus on the HCP population, especially those facing serious occupational risks of fatal disease infection (19). Given a general lack of evidence for effective PTSD treatments, especially for HCP, medical decision-making with proper information can be challenging. On the other hand, though a number of reviews focusing on the mental health of HCP are published since the COVID-19 pandemic, none focuses on PTSD (20, 21).

Research suggests that PTSD has a better prognosis if clinical interventions are applied earlier; otherwise, the individual could suffer from PTSD almost permanently, with hardly reversible stress. An early intervention would therefore be significantly beneficial to relieve the symptoms and prevent deterioration (22). As the COVID-19 global pandemic puts thousands of HCP in a life-threatening position and some of them have already developed PTSD symptoms, a rigorous systematic review of the recent PTSD intervention research is much needed (23).

## Methods

This systematic review followed the methods proposed by Cochrane Collaboration.

### Search Strategy

We searched the following databases to identify reports of randomized controlled trials (RCTs) on January 30, 2021: Ovid MEDLINE, Ovid Embase, Ovid PsycINFO, Cochrane Central Register of Controlled Trials (CENTRAL), and international trial registries *via* the trials portal of the World Health Organization (ICTRP), Clinicaltrials.gov, ProQuest PTSD Pubs, and ProQuest Dissertations & Theses Global.

We applied no restrictions based on date, language, or publication status to the searches. We also searched the gray literature (e.g., digital access to research theses).

**Figure 1:**
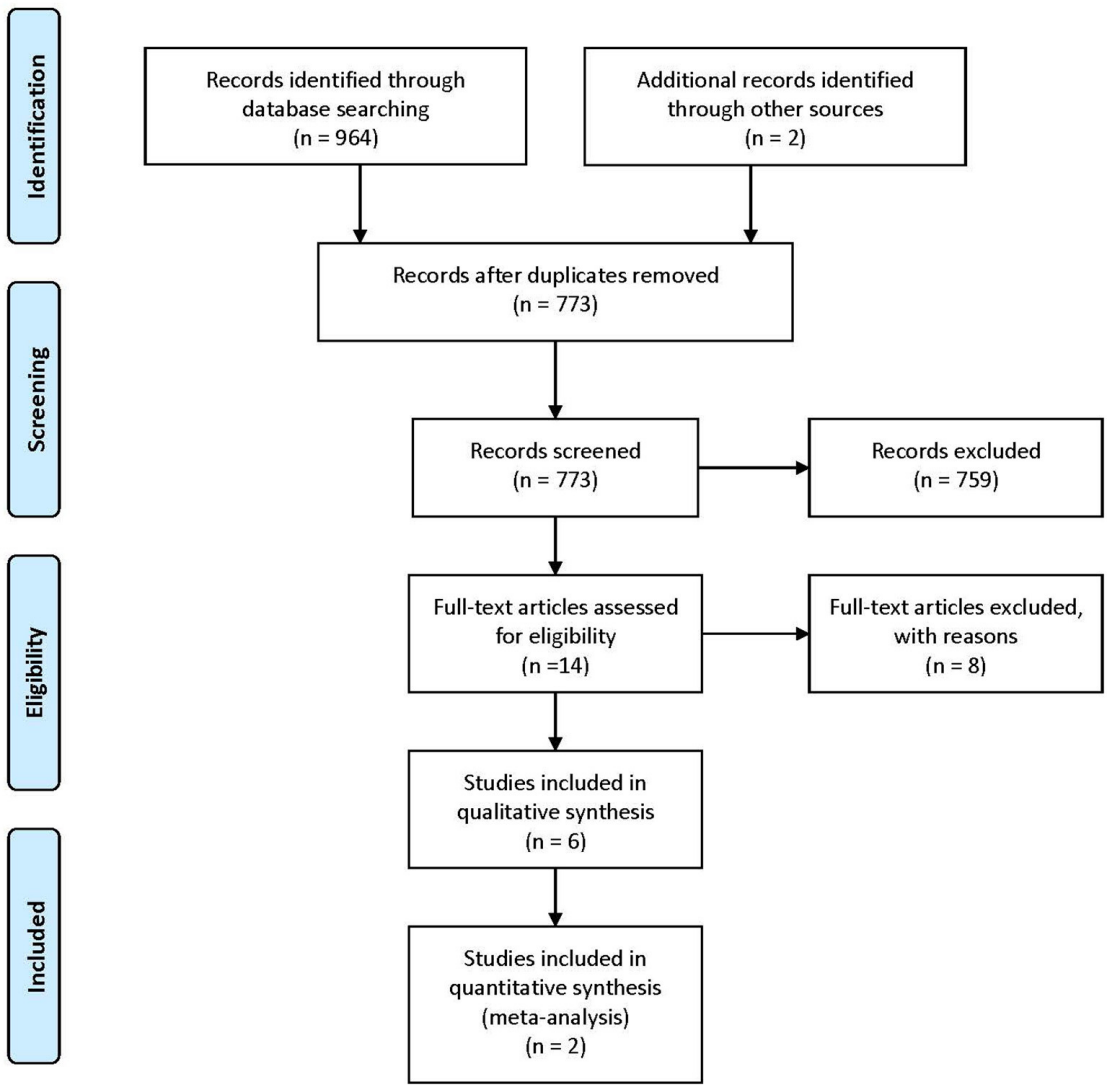
PRISMA diagram of the study selection process.

We manually searched the early editions of key journals to identify potentially relevant studies not indexed in the above-mentioned databases, contacted trialists, and subject experts for information on unpublished and ongoing studies, and requested additional trial data. We manually screened the reference lists and bibliographies of all included studies to identify other relevant references. We also contacted all authors of the included studies for any unpublished or ongoing studies or studies not otherwise identified in the search (Figure 1).

### Inclusion and Exclusion Criteria

We included all published and unpublished RCTs, cluster-randomized trials, and cross-over trials (only the first randomized phase) in any type of setting involving HCP with PTSD which meet the inclusion criteria for interventions in the corresponding studies. We included trials with only a portion of the sample meeting the above-mentioned criteria provided that the relevant data can be gained from the study report or the authors and that the effect of randomization is not affected by doing so. We included studies meeting the above-mentioned criteria irrespective of whether they report any of our outcomes expected. We excluded studies targeting participants without symptoms or diagnosis and studies that involved prophylactic intervention for PTSD.

### Data Extraction

We extracted and summarized the details of the eligible studies using a data extraction sheet. Two review authors extracted the data independently and resolved the disagreements by discussion. We included studies published in duplicate once but extracted the maximal amount of data (see Table 1).

**Table 1:** Data extraction.

**Author and year**	**Sample information**	**Methods**	**Outcomes**
Bryant et al. (24)	Setting: hospital Sample size: 100	Study design: randomized clinical trial Inclusion criteria: current or retired emergency service personnel, primary diagnosis was DSM-IV criterion for post-traumatic stress disorder (PTSD), aged between 18 and 70, adequate English and ability to follow instructions Exclusion criteria: imminent plans of suicide, psychotic disorders, or substance dependence Intervention: CBT-L or CBT-B comparison: wait-list (no treatment) Timepoints for assessment: at baseline, after 3 months (post-treatment assessment), and CBT-L and CBT-B conditions were also assessed after 6 months	Primary outcomes: PTSD symptom severity, measured by Clinician-Administered PTSD Scale (CAPS); secondary outcomes: depression severity, measured by BDI; quality of life, measured by World Health Organization Quality of Life-BREF
Nishi et al. (25)	Setting: hospital Sample size: 172	Study design: single-blind, randomized, parallel-group field trial Inclusion criteria: aged 18 years or older, a native Japanese speaker or non-native speaker with Japanese conversational abilities, physically, and psychologically capable of understanding and providing, consent for study participation Exclusion criteria: regular intake of warfarin for at least 3 months before deployment Intervention: seven capsules per day (70% DHA and 7% eicosatetraenoic acid), each containing 320 mg of fish oil Comparison: psychoeducation Timepoints for assessment: at 2, 4, 8, and 12 weeks from the start of the intervention	Primary outcomes: PTSD symptoms severity, measured by Impact of Event Scale-Revised; secondary outcomes: depression measured by Center for Epidemiologic Studies Depression Scale
Kim et al. (26)	Setting: hospital Sample size: 22	Study design: three-arm randomized controlled study Inclusion criteria: age >18, employment as a nurse at the UNM Hospital Exclusion criteria: an inability to participate in the exercise program, a positive answer to any of the seven screening questions on the Physical Activity Readiness Questionnaire, or current use of systemic glucocorticoid Intervention: mindfulness-based stretching and deep breathing exercise Comparison: not reported Timepoints for assessment: at baseline and at weeks 4, 8, and 16	Primary outcomes: PTSD symptoms severity, measured by PCL-C; secondary outcomes: none reported
Mealer et al. (27)	Setting: hospital Sample size: 27	Study design: single-center, randomized, controlled study Inclusion criteria: currently working 20 h per week at the ICU bedside, no underlying medical condition that would be a contraindication to exercise scored 82 or less on the CD-RISC Exclusion criteria: unable to participate in a 2-day educational workshop or having a medical condition that would limit exercise Intervention: 2-day educational workshop; written exposure therapy; MBSR practices; exercise; event-triggered counseling sessions Comparison: exercises, details not reported Timepoints for assessment: before the intervention started and within 1 week after the intervention ended	Primary outcomes: PTSD symptoms severity measured by The Post-traumatic Diagnostic Scale; secondary outcomes: anxiety and depression, measured by The Hospital Anxiety and Depression Scale
Ruehl (3)	Setting: hospital Sample size: 19	Study design: single-center, randomized, controlled study Inclusion criteria: currently employed as a nurse in the Hematology/Oncology, PICU or NICU at Rady Children's Hospital San Diego for at least 3 months or employed as an Emergency Room or Adult Psychiatric nurse working at the same facility for at least 3 months; hold one of the following nursing degrees: LVN, RN, ASN, BSN, or MSN; indicated at least one work-related or personal stressor; reported the experience of one or more traumatic life events, as measured by the Traumatic Life Events Questionnaire; be able to read and write in English and be able to write for the required duration of 20–30 min, on three separate occasions Exclusion criteria: current medical diagnosis of a major chronic illness (i.e., heart disease, cancer, hypertension, diabetes, HIV, liver/kidney disease); starting new medication, or with medication changes <1 month prior to study start dates; have evidenced symptoms of psychotic spectrum disorders, bipolar disorder, dissociative disorders, or organic brain damage, as indicated by a recent diagnosis, past/current hospitalizations, active psychosis, or use of antipsychotic medications; reported a current or recent suicidal ideation/threat within the past 6 months or suicidal attempt within the past year	Primary outcomes: PTSD symptoms severity, measured by Secondary Traumatic Stress Scale; secondary outcomes: depression severity, measured by BDI
		Intervention: expressive writing instructions, which allow participants the choice to write about a traumatic event or chronic stressor that was either personal or work-related Comparison: same exact protocol but instructions were modified which was strictly about activities outside of work Timepoints for assessment: at pre-test (1 week prior to the first writing session), post-test (1 week after the last writing session), and follow-up (6 weeks after the last writing session)	
Serrano-Ripol et al. (28)	Setting: hospitals, primary care centers, and care homes Sample size: 440	Study design: blinded, 2 weeks, individually randomized, parallel group, controlled trial Inclusion criteria: male and female HCWs aged >18, who report having provided healthcare to patients with COVID-19 during the viral outbreak in Spain (from the onset of the health emergency to the recruitment time). For this study HCWs will be defined as professionals regulated by a health system who deliver care and services whose primary intent is to enhance health. HCWs from any medical specialty (pneumology, internal medicine,emergency, primary care, etc.) and role (doctors, nurses, nurse assistants, etc.) with access to a smartphone will be included Exclusion criteria: HCWs with no access to a smartphone, or not able to download and activate the app used to deliver the intervention during the next 10 days following the baseline assessment in their smartphone	Primary outcomes: difference between the intervention and control groups in the mean overall score the Depression, Anxiety and Stress Scales (DASS21) instrument; secondary outcomes: difference between intervention and control groups in the mean scores of the following instruments: PTSD symptoms, measured by Davidson Trauma Scale; burnout symptoms, measured by Maslach Burnout Inventory—Human Services Survey (MBI-HSS); severity of insomnia, measured by Insomnia Severity Index (ISI); self-beliefs measured by General Self-Efficacy Scale (GSE); subjective assessments of usability, measured by System Usability Scale (SUS)

### Data Synthesis Methods

#### Risk of Bias

Two review authors independently assessed each included study using the Cochrane Collaboration tool for assessing the risk of bias, which addresses six specific domains (sequence generation, allocation concealment, etc.) (29). We completed a “risk of bias” table for each eligible study (Table 2).

**Table 2:** Summary of findings.

**Outcomes**	**Anticipated absolute effects^a^ (95% CI)**		**Relative effect**	**Number of participants**	**Certainty of the evidence**	**Comments**
	**Risk with no treatment**	**Risk with CBT-B**	**(95% CI)**	**(studies)**	**(GRADE)**	
**CBT-B compared to no treatment for HCP with PTSD**						
PTSD symptom severity assessed with CAPS	–	MD 27.8 higher	–	67	⊕⊕⊕⊖	–
Scale: from 19 to 136		(17.12 higher to 38.48 higher)		(1 RCT)	Moderate^b^	
Follow-up: 6 months						
Depression severity assessed with BDI	–	MD 13.6 higher	–	67	⊕⊕⊕⊖	–
Scale: from 0 to 63		(4.55 higher to 22.65 higher)		(1 RCT)	Moderate^b^	
Follow-up: 6 months						
**CBT-L compared to no treatment for HCP with PTSD**						
PTSD symptom severity assessed with CAPS	–	MD 26.5 higher	–	67	⊕⊕⊕⊖	–
Scale: from 19 to 136		(15.75 higher to 37.25 higher)		(1 RCT)	Moderate^b^	
Follow-up: 6 months						
Depression severity assessed with BDI	–	MD 12.5 higher	–	67	⊕⊕⊕⊖	–
Scale: from 0 to 63		(3.3 higher to 21.7 higher)		(1 RCT)	Moderate^b^	
Follow-up: 6 months						
**CBT-L compared to CBT-B for HCP with PTSD**						
PTSD symptom severity assessed with CAPS	–	MD 1.3 lower	–	66	⊕⊕⊖⊖	–
Scale: from 19 to 136		(12.13 lower to 9.53 higher)		(1 RCT)	Low^c^	
Follow-up: 6 months						
Depression severity assessed with BDI	–	MD 1.1 lower	–	66	⊕⊕⊖⊖	–
Scale: from 0 to 63		(10.17 lower to 7.97 higher)		(1 RCT)	Low^c^	
Follow-up: 6 months						
**MBX compared to no treatment for HCP with PTSD**						
PTSD symptom severity assessed with PCL-C	–	MD 17.2 higher	–	22	⊕⊕⊖⊖	–
Scale: from 17 to 85		(6.57 higher to 27.83 higher)		(1 RCT)	Low^d^	
Follow-up: 16 weeks						

a *The risk in the intervention group (and its 95% confidence interval) is based on the assumed risk in the comparison group and the relative effect of the intervention (and its 95% CI)*.

b *Downgraded one level: serious imprecision with a small sample size*.

c *Downgraded two levels: twice for very serious imprecision with a small sample size and wide CI*.

d *Downgraded two levels: twice for serious imprecision with a small sample size and once for serious risks of bias for blinding of outcome assessment*.

Two review authors independently assessed the quality of the evidence for all outcomes using the GRADE approach, applying the guidelines provided in Chapter 11.2 of the Cochrane Handbook for Systematic Reviews of Interventions (30).

According to the GRADE approach, factors that may decrease the quality level of a body of evidence were reported to complete the GRADE assessment. We considered a study as having a high risk of bias if the tools and scales used to measure the outcomes were self-reported, but we did not downgrade it as some psychological outcomes are only feasible through self-report and because the scales and measurements used were considered standard and reliable tools by the studies we included. We judged a study as having high risks of performance bias but did not downgrade it as it is impossible to blind the patients and the performer.

We downgraded if the sample size of the participants in one comparison was <100 or if the confidence intervals (CI) around effects included both appreciable benefit and appreciable harm. We downgraded if the number of unclear risks was more than three (including three). We included a “summary of findings” table (Table 2) to present the main findings.

#### Meta-Analysis

The studies were grouped according to trauma type where possible. We presented the outcome results for each trial with 95% CI. We reported estimates for dichotomous outcomes (e.g., dropout for any reason) as risk ratios (RR). We used RR rather than odds ratio (OR) since, when the event rates are high, ORs can give an inflated impression of the effect size (31). We reported outcomes relating to continuous data (e.g., reduced severity of PTSD symptoms and severity of depression) as mean differences (MD) and overall effect size (with 95% CI) where outcomes were reported on the same scale or standardized mean difference (SMD) where outcomes were reported on different scales. We obtained SMDs by calculating the difference between raw means and dividing by the pooled variance of treatment and control conditions. Where trial data is of sufficient quality and sufficiently similar, we combined data in a meta-analysis to provide a pooled effect estimate. We used a fixed-effect model in the first instance; when finding no statistical heterogeneity, we used a random-effects model to check the robustness of the fixed-effect model. Where there was substantial (over 50%) statistical heterogeneity, we reported the random-effects model only and stated so. We undertook a descriptive review of the studies that we did not include in the review.

## Results

### Description of Studies

This review included six parallel studies randomizing 810 participants, as shown in Table 1. Three studies were undertaken in the United States, one in Australia, one in Japan, and one in Spain.

The participants in the studies were health personnel with PTSD symptoms working in hospitals, emergency service personnel with PTSD, and disaster medical personnel with PTSD symptoms.

For intervention, two studies took exercises into interventions, one used a complex of interventions, another used different types of cognitive behavioral therapy (CBT), one described an expressive writing instruction, one study used a pharmacological approach, and one used a mobile-based intervention.

For primary outcomes, five studies reported PTSD symptom severity using self-reported scales; only one (24) used instructed scale during the interview, assessed by professionals.

None of the studies reported adverse events (i.e., dropout rate) for primary outcomes. In terms of secondary outcomes, four reported depression, only one reported anxiety, and one reported quality of life.

### Effects of Intervention

#### Primary Outcome: PTSD Symptom Severity

Only two of the studies which reported PTSD symptom severity were included in the meta-analysis: one focused on the long- and short-term effects of a 3-month CBT treatment, while the other took Mindfulness-Based Stretching and Deep Breathing Exercise (MBX) as an intervention for 8 weeks.

**Figure 2:**
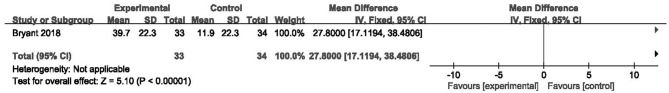
Forest plot of network meta-analysis of the end-of-treatment effect on post-traumatic stress disorder symptom severity between cognitive behavioral therapy-brief and no treatment.

A much lower level of PTSD symptom severity in the Cognitive Behavioral Therapy-Brief (CBT-B) group was reported, compared with no treatment, as assessed by a Clinician-Administered PTSD Scale (CAPS) from baseline to 3 months; the difference in means at 3 months was 27.80 (95% CI, 17.12–38.48) (Figure 2).

**Figure 3:**
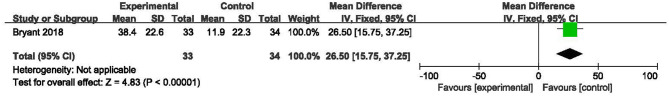
Forest plot of network meta-analysis of the end-of-treatment effect on post-traumatic stress disorder symptom severity between cognitive behavioral therapy-long and no treatment.

**Figure 4:**
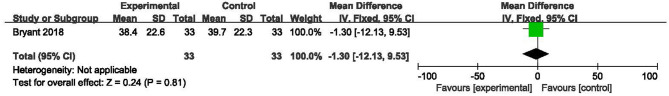
Forest plot of network meta-analysis of the end-of-treatment effect on post-traumatic stress disorder symptom severity between cognitive behavioral therapy-long and cognitive behavioral therapy-brief.

A similar effect was also reported in the Cognitive Behavioral Therapy-Long (CBT-L) group; the difference in means at 3 months was 26.50 (95% CI, 15.75–37.25) (Figure 3). These two comparisons were both moderate-certainty evidence, downgraded once for serious imprecision due to a low number of participants. The interventions probably reduce the PTSD symptom severity compared with no treatment. As for CBT-B and CBT-L, the study reported no difference in PTSD symptom severity between the CBT-L group and CBT-B group; the difference in means at 3 months was −1.3 (95% CI, −12.13 to 9.53) (Figure 4), and we considered it low-certainty evidence after downgrading once for serious imprecision and once for wide CI. We believe that CBT-L and CBT-B may have no difference in reducing the PTSD symptom severity. Additionally, the study reported that four participants became unreachable after the treatment and 14 more after follow-up. None of these dropouts were due to the adverse effects of the intervention.

**Figure 5:**
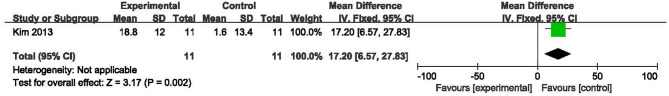
Forest plot of network meta-analysis of the end-of-treatment effect on post-traumatic stress disorder symptom severity between mindfulness-based stretching and deep breathing exercise and no treatment.

A lower level of PTSD symptom severity in the MBX group compared with no treatment was reported, assessed by PTSD Checklist–Civilian version (PCL-C) from baseline to 8 weeks. The difference in means at 3 months was 17.2 (95% CI, 6.57–27.83) (Figure 5). This is low-certainty evidence, downgraded twice for serious imprecision, once for a low number of participants, and once for a wide CI. We thus consider that the MBX may reduce the PTSD symptom severity compared with no treatment.

#### Secondary Outcome: Depression Severity

**Figure 6:**
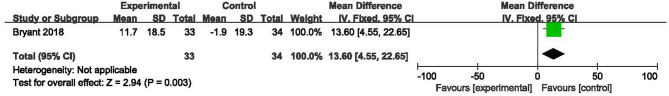
Forest plot of network meta-analysis of the end-of-treatment effect on depression severity between cognitive behavioral therapy-brief and no treatment.

**Figure 7:**
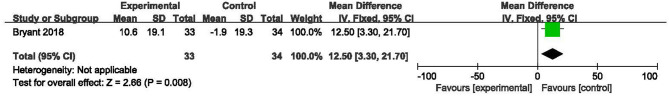
Forest plot of network meta-analysis of the end-of-treatment effect on depression severity between cognitive behavioral therapy-long and no treatment.

**Figure 8:**
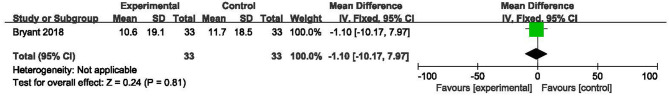
Forest plot of network meta-analysis of the end-of-treatment effect on depression severity between cognitive behavioral therapy-long and cognitive behavioral therapy-brief.

The only reported secondary outcome available for assessment was depression severity by Bryant et al. who reported a lower level of depression severity in the CBT-B group compared with no treatment, as assessed by Beck Depression Inventory—second edition (BDI) from baseline to 3 months; the difference in means at 3 months was 13.60 (95% CI, 4.55–22.65) (Figure 6). As for the CBT-L group, the difference in means at 3 months was 26.50 (95% CI, 15.75–37.25) (Figure 7). We consider these as moderate-certainty evidence, downgraded once for serious imprecision due to a low number of participants. We believe that the interventions probably reduce the depression symptom severity compared with no treatment. In comparison between CBT-L and CBT-B, the difference in means at 3 months was −1.1 (95% CI, −10.17 to 7.97) (Figure 8). We consider it low-certainty evidence, downgraded once for serious imprecision and once for wide CI. We consider that CBT-L may have no difference in reducing depression severity compared with CBT-B.

Other expected secondary outcomes (quality of life and anxiety severity) were reported in the included studies but unavailable for further analysis or GRADE assessment as necessary data was not reported.

## Conclusions

The included studies recruited 801 qualified HCP from hospitals, emergency service, and national emergency medical team and compared series of interventions, such as CBT, MBX, and resilience training, in seven comparisons.

Generally, data on many outcomes were limited and sometimes unavailable. For key outcome, only two studies reported enough data for us to calculate the most appropriate measure of MD (SD) for PTSD symptom severity and further assessed with GRADE methods. Additionally, only one study reported available data for depression severity and was further analyzed. We concluded that the most effective and feasible treatment option for HCP with PTSD is still pending. Beyond this, treatment protocols varied across studies in terms of treatment length, though these variations are common in clinical practice.

## Discussion

We found very limited reviews that evaluate the clinical efficacy of interventions in treating or preventing PTSD for HCP. We found that Lewis et al. conducted a Cochrane review in an ICU setting and a latest review focused on prevention and management from an administration perspective and reported risk factors for occupational stress of HCP (32). Our review focuses on PTSD, a widespread and severe risk faced by the HCP group in a pandemic. We draw together all relevant RCT studies that evaluated the therapeutic treatments of PTSD among HCP and collected high levels of RCT evidence following Cochrane's rigorous criteria to provide the evidence certainty of various interventions for the HCP group. The review is highly pertinent at present and may serve as a necessary protocol and reference for future pandemics.

## Limitations and Implications

The certainty of the evidence in the studies examined is usually low due to a small sample size. Moreover, whether the professionals in the studies were adequately trained to follow the standard protocols of various therapies was not clear; the actual timing and length of the interventions were not elaborated and varied from practices.

Very limited data on secondary outcome like anxiety and depression was reported for consideration in PTSD treatment for HCP despite its importance. Practitioners should take this into account, together with the evidence on PTSD symptom severity. There was a degree of heterogeneity in terms of symptom severity, types of traumatic event, and duration of symptoms, which may affect the effect of the different treatments, which the included studies did not address (33).

Since the methods used in the trials were not well-described, we were not able to examine the biases involved. Another problem found during our research is the dis-unified use of the measurement scales. Different scales administrated by professionals and participants put a different weight on different aspects of PTSD (symptom types, severity, duration, etc.), making it difficult to choose for practice. Moreover, dropouts and adverse events were not clearly reported in the studies, which pose problems for the evaluation of feasibility and the effects of intervention.

## Implications for Research

Future research should investigate the effect of PTSD intervention for HCP, given the current lack of high-quality evidence in this area. Any future studies should, alongside standard areas of good practice:

- have a standardized objective and validated PTSD assessment tools, both self-evaluated and professionally evaluated scales, to evaluate PTSD symptom severity and other characteristics of PTSD to better diagnose and monitor the improvements in a more precise and definitive way,
- have appropriate and standard follow-up times to collect maximal and sufficient information on important outcomes such as change in PTSD symptom severity as assessed by health professionals/patients: as most treatment options require uninterrupted long-term application, concordance depends on how well the treatment is perceived, especially in view of the need for a dynamic and relatively subjective psychotherapy,
- give more specific and solid evidence for adopting bundle interventions, as the combination of different interventions without evidence basis would waste resources and cause confusion, and
- collect and report outcomes such as adverse events, health-related quality of life, and cost-effectiveness.

## Data Availability Statement

The original contributions presented in the study are included in the article/supplementary material. Further inquiries can be directed to the corresponding authors.

## Author Contributions

ZS and ZL had the initial research idea. ZS, CY, and ZL formulated the research questions and designed the study. ZS and CY searched for published work, selected articles, extracted and analyzed data, and drafted the manuscript. YZ helped with searching for articles and data selection and extraction. ZS substantially contributed to designing the searches and the statistical analysis plan, writing the manuscript, and interpreting the findings. CY and YZ substantially contributed to the manuscript by providing review comments and edits. All the authors have read and approved the final manuscript. The corresponding author attests that all the listed authors meet the authorship criteria and that no others meeting the criteria have been omitted.

## Conflict of Interest

The authors declare that the research was conducted in the absence of any commercial or financial relationships that could be construed as a potential conflict of interest.

## Publisher's Note

All claims expressed in this article are solely those of the authors and do not necessarily represent those of their affiliated organizations, or those of the publisher, the editors and the reviewers. Any product that may be evaluated in this article, or claim that may be made by its manufacturer, is not guaranteed or endorsed by the publisher.
